# Detection of SNCA and FBN1 Methylation in the Stool as a Biomarker for Colorectal Cancer

**DOI:** 10.1155/2015/657570

**Published:** 2015-02-23

**Authors:** Wen-han Li, Hao Zhang, Qi Guo, Xuan-di Wu, Zi-sen Xu, Cheng-xue Dang, Peng Xia, Yong-chun Song

**Affiliations:** ^1^The First Affiliated Hospital of Xi'an Jiaotong University, Xi'an, Shaanxi 710061, China; ^2^Xi'an TB & Thoracic Tumor Hospital, Xi'an, Shaanxi 710061, China; ^3^Child & Maternity Health Hospital of Shaanxi Province, Xi'an, Shaanxi 710003, China

## Abstract

*Aim.* We examined the methylation status of SNCA and FBN1 genes in patients' paired tissue and stool samples for detection of colorectal cancer (CRC).* Patients and Methods*. 89 DNA tissue samples (normal/cancer) and corresponding stool samples were analyzed in our study. In addition, 30 stool samples were collected as healthy controls.* Results*. The methylation level of those samples was measured by methylation-specific polymerase chain reaction (MSP). The result shows that compared with the paired controls, both SNCA and FBN1 were significantly hypermethylated in CRC patients in tissue samples (*P* < 0.001). In the stool samples, hypermethylated SNCA and FBN1 were detected to be significantly higher than that in normal stool samples (*P* < 0.001). The combined sensitivity of at least one positive among the two markers in stool samples was 84.3%, with a specificity of 93.3%. In addition, our experiment suggested that the positive rates of SNCA and FBN1 in Dukes A stage were significantly higher than that of FOBT (*P* = 0.039; *P* = 0.006, resp.).* Conclusion*. We concluded that methylation testing of SNCA and FBN1 genes in stool sample may offer a good alternative in a simple, promising, and noninvasive detection of colorectal cancer.

## 1. Introduction

Colorectal cancer (CRC) is one of the commonest cancers in the Western world [1], accounting for 9% of cancer deaths in the USA in 2013 [2]. In many developing countries, like China, for example, a rapid increase in CRC morbidity has been shown in many investigations, especially in major cities where lifestyle has been deeply influenced by Western countries [3]. Among all CRC cases, approximately 95% are adenocarcinoma, but most of them are asymptomatic in their early stages. Besides, some data has shown that 5-year survival rates are over 90% for Dukes A but only 5% for Dukes D. Therefore, an ideal screening tool to detect CRC with high sensitivity and specificity has a high priority. Till now, it is generally considered among clinicians that colonoscopy represents the gold standard for CRC detection [4, 5]. But considering its invasive operations, high cost, and relatively high risk of complications, it could not satisfy the demand of CRC mass screening and could not be applied in some undeveloped regions.

Recent studies have shown that hypermethylation of CpG islands commonly exists in the neoplastic tissue, while most of them are unmethylated in normal colon mucosa of CRC patients [6, 7]. These gene alterations can be detected in patient stool, serum, or other body excretive fluids [8, 9] and therefore could be considered as a potential optimal biomarker for early detection of CRC. Previous studies have identified a set of DNA methylation markers isolated from patients stool as a user-friendly strategy and cost-effective procedure for noninvasive screening and early diagnosis of CRC [10–14]. Our research center has also completed the study about methylation of SPG20 and microRNA-34s [15, 16]. In the present work, we sought to explore the feasibility of DNA methylation status of SNCA and FBN1 as a noninvasive screening tool for CRC. Additionally, we also compared the sensitivity and specificity of those hypermethylated two genes in stool with fecal occult blood test (FOBT).

## 2. Materials and Methods

### 2.1. Collection of Tissue and Stool Samples

In order to reduce bias, we designed this experiment as a blinded assay. All sample collection and preservation were taken care of by a person who did not participate in the follow-up studies. Patients with sporadic CRC who participated in this study were recruited consecutively from February 2012 to January 2014. CRC tissue and normal mucosa tissue (>10 cm away from tumor) samples were collected during surgery from 89 patients at the First Affiliated Hospital of Xi'an Jiaotong University College of Medicine (Xi'an, China). Paired fecal specimens were collected in a 15 mL collection tubes before bowel cleansing. In the meantime, another 30 stool samples from healthy individuals were also obtained. All individuals underwent a colonoscopy with comprehensive examination of the right and left colon and the rectum, which was performed by experienced gastroenterologists using the same preps. Patients with prior colorectal resection and history of any cancer or chemotherapy or radiation therapy were excluded from the study. All samples were immediately frozen and stored at −80°C until DNA was extracted. In order to reduce bias, samples were randomly coded before processing. All patients voluntarily joined this study with written informed consents to have their biologic specimens analyzed. This study was announced by the Ethical Committee of the First Affiliated Hospital of Xi'an Jiaotong University.

### 2.2. DNA Isolation

DNA was extracted from colorectal tissues (10 ± 1 mg) with the TIANamp Genomic DNA kit and for stool samples (200–220 mg) by use of QIAamp DNA Stool Mini Kit (Qiagen). All procedures were strictly carried out according to the manufacturer's instructions. The concentration of DNA was measured by ultraviolet spectrophotography and the quality of DNA was tested by PCR amplification of the human *β*-actin.

### 2.3. Bisulfite Modification

As to bisulfite genomic DNA modification, 1000 Ng of DNA was modified by EpiTect Bisulfite Kit (Qiagen) to convert all unmethylated cytosine to uracil. The bisulfite-treated DNA was eluted in 15 mL of TE buffer and stored at −20°C until being processed.

### 2.4. Methylation-Specific Polymerase Chain Reaction (MSP)

After the bisulfite treatment, we used methylation-specific PCR to testify the methylation status of the SNCA promoter. The primers specific to methylated and unmethylated sequences were shown in Table 1. The procedures are as follows: 1 *μ*L bisulfite-modified DNA was amplified in a total volume of 25 *μ*L containing 1× PCR buffer (Takara), 200 *μ*M dNTPs, 0.4 *μ*M concentration of each primer (BGI), and 1 U of HotStarTaq enzyme (Takara). PCRs were performed as the following conditions: an initial denaturation at 95°C for 10 min, followed by 35 cycles of denaturation at 94°C for 30 s, annealing at 53°C (SNCA) or 48°C (FBN1) for 30 s and 72°C for 30 s, and a final extension at 72°C for 5 min.

We used water without DNA as a negative control. Product was visualized by electrophoresis in a 2% agarose gel and analyzed by a gel imaging system. The methylation pattern result was judged by the distribution of visible bands.

### 2.5. Fecal Occult Blood Testing (FOBT)

FOBT was performed by a single immunochemical FOBT (IFOBT) with Magstream Hem Sp, an immunochemical measurement of hemoglobin. The tests were done independently at the clinical laboratory in the First Affiliated Hospital of Xi'an Jiaotong University College of Medicine.

### 2.6. Statistical Analysis

In the present study, associations between variables were calculated using Fisher's exact test or chi-square test. Statistical analyses were performed with the SPSS 13.0 software. *P* values <0.05 (two-sided) were considered statistically significant.

## 3. Results

In order to identify DNA methylation biomarker potentially suitable for early diagnosis of colorectal cancer, we extracted 208 DNA samples from 89 patients with histologically diagnosed CRC and 30 healthy controls (89 CRC tissue samples, 89 normal mucosa tissue samples, 89 CRC stool samples, and 30 healthy control stool samples). There was no significant difference with respect to age and gender between cases and controls (age: *P* = 0.993; gender: *P* = 0.124). All DNA samples could be successfully modified with sodium bisulfite and amplified by MSP.

**Figure 1:**
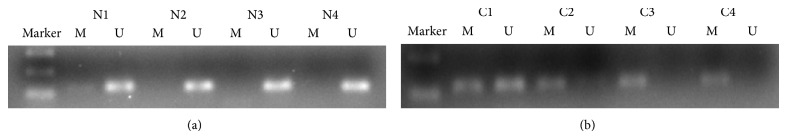
Detection of unmethylated (U) and methylated (M) SNCA in tissue of normal mucosa (N1–N4) and colorectal cancer (C1–C4).

**Figure 2:**
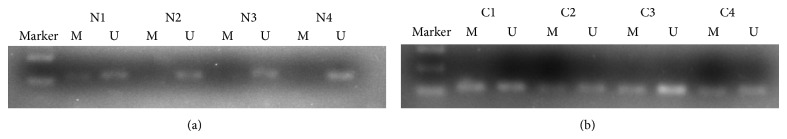
Detection of unmethylation status (U) and hypermethylation status (M) of SNCA in stool samples of patients with stool samples of healthy individuals (N1–N4) and colorectal cancer (C1–C4).

**Figure 3:**
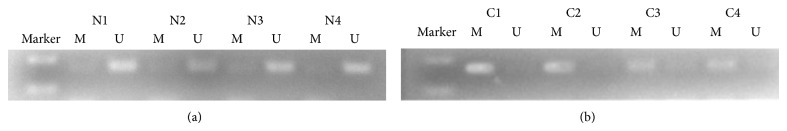
Detection of unmethylated (U) and methylated (M) FBN1 in tissue of normal mucosa (N1–N4) and colorectal cancer (C1–C4).

**Figure 4:**
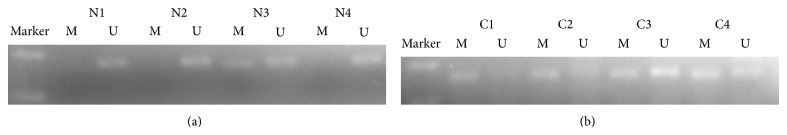
Detection of unmethylation status (U) and hypermethylation status (M) of FBN1 in stool samples of patients with stool samples of healthy individuals (N1–N4) and colorectal cancer (C1–C4).

Hypermethylation of SNCA gene was detected from 64 of 89 CRC tissue samples (71.9%) and 1 of 89 matched normal mucosa tissue samples (1.1%). For FBN1, methylation was found in 77.5% (69/89) of tumor tissue samples and 3.4% (3/89) of normal mucosa. The data indicates that hypermethylation status between cancer tissue and nonneoplastic tissue was significantly different (*P* < 0.001; *P* < 0.001) (Figures 1 and 3; Table 2). We next analyzed methylated SNCA and FBN1 in stool DNA. The frequency of methylated SNCA in stool samples reached 70% (62/89) for CRC, and none of 30 healthy volunteers stool samples were detected as methylation status (*P* < 0.001) (Figure 2; Table 2). For FBN1, 70.8% (63/89) of CRC stool samples were methylated, which is significantly higher than that for normal individuals (6.7%, 2/30) (Figure 4; Table 2). It indicated that the sensitivity for screening CRC using detection of SNCA and FBN1 methylation in stool DNA by MSP was 70% and 70.8%, respectively. The specificity is 100% and 93.3%, respectively. In addition, 75 out of 89 (84.3%) CRC stool samples were hypermethylated in at least one of the two analyzed markers, in contrast to 2 of the 30 (6.7%) healthy controls (*P* < 0.01) (Table 3). The result shows that comethylation of the two genes reaches 84.3% sensitivity and 93.3% specificity. We next explored the correlation between clinicopathological data and methylation status of DNA in stool samples, and the result was shown in Table 4. No correlation was found of overall methylation with age, gender, tumor location, pathological pattern, or Dukes' stage. Then we compared the diagnostic value between gene's methylation and FOBT in early CRC. The sensitivity of the MSP assay for CRC was significantly higher than that of FOBT in Dukes A stage (SNCA: 64.7% versus 29.4%, *P* = 0.039; FBN1 76.5% versus 29.4%, *P* = 0.006) (Table 2). Thus, both SNCA methylation and FBN1 methylation in stool were indicated to be more sensitive compared to FOBT in the early stage of CRC.

## 4. Discussion

For sporadic CRC, the accumulation of genetic and epigenetic alterations is increasingly recognized as a crucial process that induce colonic epithelial cells into colon adenocarcinoma cells [17–19]. The epigenetic silencing of tumor suppressor genes has been considered as one of the principal mechanisms that lead to tumor's gradual progression [20, 21]. Researchers have found that the aberrant methylation of the cytosine residues of CpG-rich sequences (CpG islands) which located within the promoter regions of genes induced the transcriptional silencing of tumor suppressor genes. Those genes regulate basic functions of cell cycle such as proliferation, apoptosis, and DNA repair. The methylation states could be detected in body fluids and easily measured by PCR-based methods with a high sensitivity [22]. Therefore, stool DNA testing could be of great clinical value in providing a more attractive alternative tool for early CRC detection.

In Lind et al.'s study, promoter of the SNCA gene was found frequently hypermethylated in colorectal cancer tissue samples, whereas normal mucosa samples were rarely methylated [23]. Her research exhibited that among 74 patients in CRC test set, 54 showed a methylated SNCA promoter as detected by MSP (73%), in contrast to none of 51 healthy controls subjects (*P* < 0.001). To further assess the clinical value of methylated SNCA in CRC, we analyzed fecal DNA from stool samples of 89 CRC individuals and 30 healthy controls. The result indicated 70% sensitivity and 100% specificity, which was a little lower than in tissue samples (71.9% sensitivity). In our previous studies, we have proved that the promoters of FBN1 gene are frequently hypermethylated in patients with colorectal tumors and can be detected in their stool samples [24]. On the basis of our pervious experiment, we continued the study by extending the sample volume (SNCA and FBN1 genes were detected at the same group of samples). The result showed that methylated FBN1 was detected in 77.5% (69/89) of CRC tissue samples and 70.8% (63/89) of stool samples, which was similar to our pervious study (CRC tissue samples: 78.7%; CRC stool samples: 72%). The combined sensitivity of at least one positive between the two markers reached satisfactory outcome with 84.3% in tumor stool samples (75/89). In addition, some reports found that though FOBT is the only available noninvasive screening method for CRC at present, it has relatively low sensitivity, especially for early stage cancer [25, 26]. In accordance with the early published data, our study found that only 29.4% (5/17) early CRC was detected, suggesting that detection of methylated gene in fecal DNA can be a promising biomarker for early detection of CRC.

The current methods of detecting CRC that are used in clinical practice are FOBT, colonoscopy, and serum tumor markers. FOBT is the only available noninvasive screening method at present and yet has a relatively low sensitivity. Colonoscopy is more sensitive alternative, but it needs complicated bowel preparation, and a small but nonnegligible risk of major complications exists. Theoretically, molecule marker should be more specific than protein biomarker such as carcinoembryonic antigen (CEA), because the former is shed from tumor cells and could be amplified by PCR methods to produce a strong signal, but the latter could be expressed in more than one type of cancer and easily influenced by other factors [27]. Even though protein biomarkers are still widely used in clinical practice today, we believe they will eventually be replaced by genetic diagnosis for its low specificity. According to Wang and his colleague's research [27], the procedure of detecting methylation in stool sample has not yet been standardized, so it is relatively difficult to compare different biomarkers under different experimental circumstances. For example, the buffer we use to isolate and store DNA is different in each study, and we cannot calculate the degradation loss of DNA during transport and storage. So, it is imperative to set up a standard guideline about the procedure of methylation to improve the comparability between various results.

Although the result shows that comethylation of the two genes reached a relative high sensitivity, there are still 15.7% (14/75) of missing CRC for a diagnostic test; this phenomenon may be caused by the existence of the so-called CpG island methylator phenotype-negative tumors [28, 29] or incomplete bisulfite modification. We may improve the sensitivity through combination of different methylated biomarkers. Our next step is to detect genes together (SPG20, FBN1, microRNA-34s, and SNCA) to explore a method that could reach a greater precision for the early detection of CRC.

## 5. Conclusions

In summary, methylation of SNCA and FBN1 gene has a relatively high sensitivity and specificity in the detection of CRC; what is more, it may serve as a promising predictive marker for the noninvasive screening for CRC.

## Figures and Tables

**Table 1:** Methylation-specific primers of SNCA and FBN1.

Primer set	Forward primer	Reverse primer	Amp size (bp)	Annealing temperature (°C)
SNCA-M	CGGGTTGTAGCGTAGATTTC	CGTCGAATAACCACTCCC	125	53
SNCA-U	GTGTGGGTTGTAGTGTAGATTTT	TCATCAAATAACCACTCCCAA	129	53
FBN1-M	GTATTTTTTTCGCGAGAAATC	AATCGTAACCGCTACAACC	164	48
FBN1-U	AAAGTATTTTTTTTGTGAGAAATT	CCCAATCATAACCACTACAACC	170	48

**Table 2:** The positive rate of hypermethylated SNCA and FBN1 in tissue and stool samples.

Parameters	SNCA methylation		Positive percent	*P* value	FBN1 methylation		Positive percent	*P* value
	Positive	Negative			Positive	Negative		
Tissue samples								
CRC	64	25	71.9%	<0.001^*^	69	20	77.5%	<0.001^*^
Normal	1	88	1.1%		3	89	3.4%	
Stool samples								
CRC	62	27	70%	<0.001^*^	63	26	70.8%	<0.001^*^
Normal	0	30	0%		2	28	6.7%	
Stool samples (Dukes A stage)								
SNCA methylation	11	6	64.7%	0.039^*^	13	4	76.5%	0.006^*^
FOBT	5	12	29.4%		5	12	29.4%	

Using chi-square for this statistic.

^*^Statistically significant.

**Table 3:** The positive rate of at least one hypermethylated gene in stool samples.

	SNCA + FBN1 methylation		Positive percent	*P* value
	Positive	Negative		
CRC	75	14	84.3%	<0.001^*^
Normal	2	28	6.7%	

When the markers were used in combination, the test was considered to be positive if one marker reached the threshold and negative if both markers were negative.

Using chi-square for this statistic.

^*^Statistically significant.

**Table 4:** Correlation between SNCA and FBN1 hypermethylation status in stool DNA of CRC patients and clinicopathological parameters.

Parameters	Number of cases	SNCA		FBN1	
		Methylation	*P* value	Methylation	*P* value
Age					
<50	24	17 (70.8%)	0.958^a^	19 (79.2%)	0.173^a^
50–60	24	18 (75.0%)		17 (70.8%)	
60–70	22	15 (68.2%)		15 (68.2%)	
>70	19	14 (73.7%)		18 (94.7%)	
Gender					
Male	54	38 (70.4%)	0.688^a^	41 (75.9%)	0.653^a^
Female	35	26 (74.3%)		28 (80.0%)	
Tumor location					
Left hemicolon	19	13 (68.4%)	0.470^a^	17 (89.5%)	0.255^a^
Transverse colon	5	4 (80.0%)		5 (100%)	
Right hemicolon	8	4 (50.0%)		6 (75.0%)	
Rectum	57	43 (75.4%)		41 (71.9%)	
Pathological pattern					
Ulcerative type	60	42 (70.0%)	0.581^a^	46 (76.7%)	0.504^a^
Protrude type	24	19 (79.2%)		20 (83.3%)	
Infiltrating type	5	3 (60.0%)		3 (60.0%)	
Dukes' stage					
A	17	11 (64.7%)	0.323^a^	13 (76.5%)	0.237^a^
B	36	29 (80.6%)		25 (69.4%)	
C	36	24 (66.7%)		31 (94.4%)	

^a^Using chi-square for this statistic.
